# Evaluation of the responsiveness of outcome measures after spine injection: A retrospective study

**DOI:** 10.1371/journal.pone.0211763

**Published:** 2019-02-27

**Authors:** Jiwoon Seo, Joon Woo Lee, Yusuhn Kang, Eugene Lee, Joong Mo Ahn, Dong Hyun Kim, Heung Sik Kang

**Affiliations:** 1 Department of Radiology, Seoul National University Bundang Hospital, Seongnam-si, Gyeonggi-do, Korea; 2 Department of Radiology, Seoul National University Boramae Medical Center, Seoul, Korea; Harvard Medical School, UNITED STATES

## Abstract

Discrepancies in patients’ responses to various outcome measures challenge clinicians’ evaluation of treatment outcomes. Therefore, we aimed to 1) evaluate the concordance of outcome measures after spine injection, 2) determine the patient variables that lead to discordant responses, and 3) suggest practical outcome measure for spine injections with good responsiveness. From October 2014 to November 2014, 164 patients with neck or low back pain who visited our outpatient clinics and had spine injections on the previous visit were enrolled. We asked patients to report changes in their symptom in the form of outcome measures: numeric rating scale, Oswestry disability index, neck disability index, residual symptom percentage and global perceived effect. The responses were categorized into three groups according to the degree of change; not improved, minimally improved, and significantly improved. The concordances of these categorized answers were evaluated. When “significantly improved” was considered as true improvement, 46 (28%) of the 164 patients had discordant responses to the four measures. There was no significant patients’ variable that affects discordance in the outcome measures. Good agreement was shown between the global perceived effect and residual symptom percentage, while the Oswestry disability index had poor agreement with the other measurements. The calculated numeric rating scale and residual symptom percentage also had low levels of agreement. However, patients with severe pre-treatment pain tended to have better agreement. In conclusion, this result suggest that the residual symptom percentage may be a more practical for clinicians and better represent patients’ improvements after spine injection.

## Introduction

Low back pain and neck pain are common causes of disability in middle- to old-aged individuals. Since only 10% to 15% of these patients require surgery or are eligible for surgery, conservative management is recommended in most patients. Conservative management may include oral medications, image-guided injections, exercise, or physical therapy. Among these, spine injections have been reported to be effective in localizing and managing low back and neck pain [1, 2].

As pain is a subjective symptom, its assessment is complicated. Various outcome measures have been developed to measure pain. Some tools are intended to rate the intensity of pain itself, e.g., the numeric rating scale and visual analog scale, while other tools are intended to assess functional status, e.g., the Oswestry disability index, neck disability index, and Roland Morris disability questionnaire. Clinicians are interested in the effectiveness of their treatment; surgery, injections or medications. However, it always been their task to measure improvement and decide the next step.

There have been several studies that evaluated or compared the responsiveness, an instrument’s ability to detect change over time, of various outcome measures. These studies often conclude that these measures have good reliability and responsiveness [3–5]. However, most of these studies evaluated patients after surgery or rehabilitation. Only a few studies have evaluated the responsiveness of specific outcome measures after spine intervention [6–9].

In clinical practice, it is often observed that some cases show confounding results after spine injection. For example, some patients have told us that their symptoms got much better after spine injections; likewise, the global perceived effect and residual symptom percentage were improved. However, their post-injection pain intensity or functional scores, such as the numeric rating scale and Oswestry disability index, were not decreased when compared to their pre-injection values. We hypothesized that discordance in these results may be due to properties of the outcome measurements or patient variables, such as age or sex. The residual symptom percentage is an easily asked and answered measure in clinical practice, while disability or functional score measurements require more time and effort. The residual symptom percentage has grossly good agreement with the global perceived effect in our experience. However, only a few studies have reported the responsiveness and reliability of self-reported pain reduction and compared it with calculated pain reduction [6, 8, 10]

Therefore, the purposes of our study were to 1) evaluate the responsiveness of outcome measures after spine injection, 2) determine the patient variables that lead to discordance in outcome measurements, and 3) suggest practical outcome measure for spine injections with good responsiveness.

## Materials and methods

### Patient selection

This retrospective study was approved by the institutional review board, and the requirement of informed consent was waived. From October 2014 to November 2014, 605 patients visited our outpatient pain clinic. Among them, 189 patients who received a spine injection during their previous visit were included in the study. The inclusion criteria were: (1) patients who visited our clinic for either neck or low back pain with or without radiculopathy, (2) those who received spine injections at the time of their last visit, and (3) patients who completed the self-reported questionnaire at the time of the visit for their pain evaluation.

Twenty-five patients were excluded based on the following exclusion criteria: patient who had (1) complaint of both back and neck pain (n = 6), (2) recurred symptom more than one year after the previous injection (n = 1), and (3) incomplete self-reported questionnaires or missing medical records (n = 18). As a result, one-hundred and sixty-four patients (men: women ratio = 85: 79; mean age = 63.3 years, standard deviation = 1.06 years, range = 22–89 years) were enrolled in this study.

### Outcome measurements

Every patient was asked to complete a set of written questionnaires to record a self-reported pain and disability measures, before visiting clinicians for interview. First, the patients scored their pain intensity with the numeric rating scale ranging from 0 to 10, with 0 indicating no pain and 10 indicating the worst pain imaginable. For the patients with back pain, a degree of disability due to back pain was measured using the translated Korean version of the Oswestry disability index, which is based on the Oswestry disability index, version 2.0. This measure consists of nine sections and has a total score of 45, as sexual life was excluded from the original version. Oswestry disability index measures a degree of disability in everyday life with nine items including walking, sitting, standing, sleeping, etc., while the item related to sexual life was excluded from the original version. Each item was scored with scale ranging from 0 to 5, while 0 indicating the least amount of disability and 5 indicating the most severe disability. On the other hand, a degree of disability due to neck pain was measured using the translated Korean version of the neck disability index. Similar to Oswestry disability index, neck disability index measures a degree of disability in ten items, which includes lifting, reading, working, driving, etc., and also scored with scale of 0 to 5. During the interview, each patient was asked to answer two additional measures. One was the global perceived effect, which is a self-assessed 5-point scale (1 = no pain, 2 = much improved, 3 = slightly improved, 4 = no change, and 5 = aggravated), While the other measure was residual symptom percentage, which is the remaining percentage of the symptom when considering the symptoms before injection as 100%.

### Spine injections

During the interview, the radiologist clearly identified the affected nerve root or segment using clinical findings and imaging studies, and determined the method of approach for the spine injection; epidural, transforaminal or facet joint injection. All spine injections were performed under biplane fluoroscopic guidance by one of four radiologists with 10 years, 4 years, 1 year, and 1 year of experience with spine injections.

### Data analysis

#### Categorization of improvement

For fair comparison between the different outcome measures, the collected samples of numeric rating scale, Oswestry disability index, and neck disability index are converted into a tractable form by leveraging following formula.

$$Converted\;measure=\frac{post-injection\;score}{pre-injection\;score}\times 100.$$

Notice that the converted measure indicates the portion of residual pain or a degree of disability after the injection. Now, to evaluate the concordance among the outcome measures, all the five outcome measures were categorized into three groups: “not improved”, “minimally improved”, and “significantly improved”. The numeric rating scale, Oswestry disability index, and neck disability index were categorized according to converted residual pain or disability based on equation above: converted measure percentage greater than 70% was considered “not improved”, between 70% to 50% was considered “minimally improved”, and less than 50% was considered “significantly improved”. This categorization was based on previously proposed ‘minimally important changes’ by Ostelo et al [11]. The residual symptom percentage was categorized as follows: more than 70% residual symptom was considered “not improved,” 50% to 70% residual symptom was considered “minimally improved”, and less than 50% residual symptom was considered “significantly improved”. When categorizing the global perceived effect, no change and aggravation were considered “not improved”, slightly improved was considered “minimally improved”, and no pain or much improved were considered “significantly improved”.

### Concordance of the measurements

**Fig 1:**
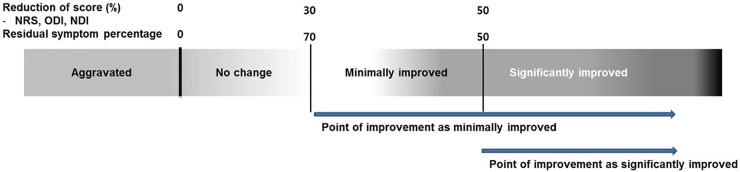
Schematic diagram of concordance evaluation. We set a decision point of improvement as either “minimally improved” or “significantly improved”. NDI, neck disability index; NRS, numeric rating scale; ODI, Oswestry disability index; RSP, residual symptom percentage.

Fig 1 is schematic diagram of how evaluation that were performed. We set a decision point of improvement as either “minimally improved” or “significantly improved”. Then, we evaluated concordance of improvement, whether patient have been improved or not.

### Statistical analysis

Paired t-tests were used to compare datasets obtained at the initial evaluation to those obtained at the follow-up after the injection. Analysis of variance (ANOVA) was conducted to compare the means of the measures indicating different levels of improvement to the global perceived effect. Chi-square tests were performed to compare sex and previous experience of a spine injection between groups categorized based on concordance. ANOVA was conducted to compare differences in the time interval between the two visits and the patients’ ages.

The intraclass correlation coefficient was used to determine the level of inter-measure agreement for improvement after the injection according to the previously mentioned categorization of improvement.

The concordance correlation coefficient was used to determine the level of agreement between the converted numeric rating scale change and residual symptom percentage. A visual representation of the data was created using a Bland-Altman plot.

All statistical analyses with the exception of the concordance correlation coefficient calculation and the Bland-Altman graph were performed using Statistical Package for the Social Sciences (SPSS) version 20.0 for Microsoft Windows (SPSS Inc.; Chicago, IL). The concordance correlation coefficient calculation and plotting of the Bland-Altman graph were performed using MedCalc (MedCalc Software; Mariakerke, Belgium). Statistical significance was defined as P < 0.05.

## Results

Among 164 patients, 122 had low back pain with or without lumbar radiculopathy, and they underwent spine injection at the lumbar level. Forty-two patients complained of neck pain with or without cervical radiculopathy, and they received a spine injection at the cervical level. The median interval between the patient visit after injection was 56 days (interquartile range: 73.41–99.34 days).

### Outcome measures before and after the spine injection

Outcome measures before and after the spine injection are summarized in Tables 1 and 2. The neck disability index and the numeric rating scale scores (p = 0.003) were significantly different following the spine injection. No differences was found in the Oswestry disability index score (p = 0.275).

**Table 1:** Mean and difference of outcome measure before and after the spine injection in neck pain.

Outcome measure	Pre-injection	Post-injection	Difference	p-value
	mean	mean	mean (95% CI)	
**NRS**	6.67	5.64	1.02 (0.38–1.67)	0.002
**NDI**	16.33	13.33	3.00 (1.13–4.87)	0.003

NDI, neck disability index; NRS, numeric rating scale; ODI, Oswestry disability index.

**Table 2:** Mean and difference of outcome measure before and after the spine injection in lower back pain.

Outcome measure	Pre-injection	Post-injection	Difference	p-value
	mean	mean	mean (95% CI)	
**NRS**	7.72	6.80	0.42 (0.08–0.76)	0.017
**ODI**	18.09	17.47	0.62 (-0.50–1.75)	0.275

NRS, numeric rating scale; ODI, Oswestry disability index.

### Changes in scores based on the global perceived effect

The converted residual pain or disability index were significantly different between patients’ global perceived effect, in each of the outcome measure, except for Oswestry disability index (Tables 3 and 4). The averages of converted score had a tendency to increase, which implied increase of residual symptom, as patient perception of pain gets worse. The exception was the neck disability index, which exhibited a reversed score change in patients in the ‘no change’ and ‘aggravated’ groups.

**Table 3:** Converted measures after spine injection in neck pain patient based on global perceived effect^a^.

Global perceived effect	N	NRS^b^	NDI^b^	RSP^b^
**No pain**	1	0.0(-)	0.0(-)	5.0(-)
**Much improved**	15	77.5(48.2–106.9)	58.7 (41.6–75.8)	30.3 (20.7–40.0)
**Slightly improved**	12	85.3 (73.8–96.9)	88.4 (70.5–106.3)	75.0(68.1–81.9)
**No change**	12	91.4(78.5–104.3)	110.5 (83.4–137.6)	98.3(94.7–102.0)
**Aggravated**	2	191.7 (-761.2–1144.63)	92.2(-131.9–317.2)	135.0(-55.6–325.6)
**Total**	42			

Note—Data show mean of score change and its 95% confidence interval.

NDI, neck disability index; NRS, numeric rating scale; RSP, residual symptom percentage.

^a^Converted measure = [post-injection] / [pre-injection score] x100

^b^*P* value less than 0.05.

**Table 4:** Converted measures after spine injection in low back pain patient based on global perceived effect.

Global perceived effect	N	NRS^b^	ODI	RSP^b^
**No pain**	0	- (-)	- (-)	- (-)
**Much improved**	30	86.4(70.5–102.2)	90.4(74.1–106.6)	35.8(29.6–42.0)
**Slightly improved**	50	96.9 (89.4–104.4)	106.6(86.6–126.6)	72.2(68.4–76.0)
**No change**	37	107.0(97.2–116.7)	116.7(88.1–169.8)	100(-)
**Aggravated**	5	117.0(84.3–149.7)	128.9(95.2–118.0)	138.0(89.6–186.4)
**Total**	122			

Note—Data show mean of score change and its 95% confidence interval.

NDI, neck disability index; NRS, numeric rating scale; RSP, residual symptom percentage.

^a^Converted measure = [post-injection] / [pre-injection score] x100

^b^*P* value less than 0.05.

### Categorized improvement of the outcome measures

The outcome measures have different percentages of improvement (Tables 5 and 6). Global perceived effect and residual symptom percentage had higher percentages of improvement than the Oswestry disability index, neck disability index, and numeric rating scale.

**Table 5:** Categorization of improvement of outcome measures in neck pain patients.

Outcome Measure	Not improved	Minimally improved	Significantly improved
**NDI**	27 (64.3)	6 (14.3)	9 (21.4)
**NRS**	29 (69.0)	7 (16.7)	6 (14.3)
**GPE**	14 (33.3)	12 (28.6)	16 (38.1)
**RSP**	19 (45.2)	12 (28.6)	11 (26.2)

Note—Data show number of case and its percentage.

GPE, global perceived effect; NDI, neck disability index; NRS, numeric rating scale; RSP, residual symptom percentage.

**Table 6:** Categorization of omprovement of outcome measures in low back pain patients.

Outcome Measure	Not improved	Minimally improved	Significantly improved
**NDI**	102 (83.6)	12 (9.8)	8 (6.6)
**NRS**	107 (87.7)	14 (11.5)	1 (0.8)
**GPE**	42 (34.4)	50 (41.0)	30 (24.6)
**RSP**	67 (54.9)	36 (29.5)	19 (15.6)

Note—Data show number of case and its percentage.

GPE, global perceived effect; NRS, numeric rating scale; ODI, Oswestry disability index; RSP, residual symptom percentage.

### Concordance of improvements in outcome measures

Tables 7–9 show the number of patient who answered concordant responses and difference in their variables, when “minimally improved” and “significant improved” set as a decision point of improvement, consecutively. If “minimally improved” is decision point of improvement, patient with converted residual pain or disability less than 70% considered improved; if “significantly improved” is decision point of improvement, patient with converted residual pain or disability less than 50% considered improved. When considering “significantly improved” as improvement in the symptom, the outcome measures of the 117 patients were concordant with the four measures (Table 7). When ‘minimally improved’ was considered as improvement, the outcome measures of 63 patients were concordant with the four measures.

**Table 7:** Concordant response on improvement.

	Decision point^a^	
Concordant response^b^	Minimally improved	Significantly improved
**2**	46	19
**3**	55	28
**4**	63	117

^a^Decision point of improvement. If “minimally improved” is decision point of improvement, patient with converted residual pain or disability less than 70% considered improved; if “significantly improved” is decision point of improvement, patient with converted residual pain or disability less than 50% considered improved.

^b^Number of concordant answer to categorized outcome measures; numerical rating score, Oswestry disability Index, neck disability index, global perceived effect, residual symptom percentage.

**Table 8:** Patient factors and concordance of the response when “minimally improved” considered as improvement.

	Number of concordant response^a^		
Patient variables	2	3	4
**Previous injections**^b^			
**yes**	33 (71.7)	30 (54.5)	34 (54.0)
**no**	13 (28.3)	25 (45.5)	29 (46.0)
**Sex**			
**Male**	24	23	38
**Female**	22	32	25
**Improvement**			
**Not improved**		30	50
**Improved**		25	13
**Total**	46 (28.0)	55 (33.5)	63 (38.4)
**Time interval** ^c^	88.24 (65.30–111.18)	89.53 (62.50–116.55)	82.52(64.30–100.75)
**Age** ^c^	62.76 (58.52–67.00)	65.11(61.89–68.33)	62.11(58.44–65.78)

Note—Data show number of cases and percentage or mean.

^a^Number of concordant answer to categorized outcome measures; numerical rating score, Oswestry disability Index, neck disability index, global perceived effect, residual symptom percentage.

^b^Experience of more than a single spine injection.

^c^Data show mean and its 95% confidence interval.

**Table 9:** Patient factors and concordance of the response, when “significantly improved” considered as improvement.

	Number of concordant response to the outcome measures^a^		
Patient variables	2	3	4
**Previous injections**^b^			
yes	13(68.4)	18 (64.3)	66 (56.4)
no	6 (31.6)	10 (35.7)	51 (43.6)
**Sex**			
Male	15	15	55
Female	4	13	62 ^c^
**Improvement**			
Not improved		6	106
Improved		22	11
Total	19 (11.6)	28 (17.0)	117 (71.3)
**Time interval**^d^	119.42 (60.35–178.49)	59.75 (34.25–85.25)	87.52 (73.23–101.81)
**Age**^d^	64 (57.20–70.80)	62.67 (57.62–67.72)	63.33(60.81–65.86)

Note—Data show number of cases and percentage or mean.

^a^Number of concordant answer to categorized outcome measures; numerical rating score, Oswestry disability Index, neck disability index, global perceived effect, residual symptom percentage.

^b^Experience of more than a single spine injection

^c^ *P* value less than 0.05

^d^Data show mean and its 95% confidence interval.

The effects of a previous injection experience (other than the injection patient had on their last visit), sex, age, and time interval to concordance are described in Tables 8 and 9. Among the 46 patients who had only two concordant responses to the outcome measures (Table 8), 33 (71.7%) patients had a previous history of receiving a spine injection. Patients who had less concordance with the outcome measures had previously received spine injections, although this finding was not statistically significant. When “significantly improved” was considered true improvement, 62 of 79 female patients had 4 concordant answers to the outcome measures (Table 9). This finding was significantly different when compared to that for male patients. The time interval between the two visits to the outpatient clinic and the patients’ ages were not significantly different.

### Agreement between converted measures and global perceived effect

The intraclass correlation coefficients of the measurements are summarized in Table 10. The intraclass correlation coefficient was 0.930 and 0.890 between the global perceived effect and the residual symptom percentage, in patient with neck and low back pain, consecutively, which was highest among the measure comparisons. However, there was poor agreement between the Oswestry disability index and the other measures.

**Table 10:** Intraclass correlation coefficient between outcome measures.

Combination of outcome measures	Intraclass correlation coefficient
**Neck pain**	
**NRS-NDI**	0.716
**NRS-GPE**	0.596
**NRS-RSP**	0.697
**GPE-NDI**	0.734
**GPE-RSP**	0.930
**RSP-NDI**	0.765
**GPE-NRS-RSP**	0.830
**GPE-NRS-NDI**	0.766
**NRS-RSP-NDI**	0.801
**GPE-RSP-NDI**	0.870
**GPE-NRS-RSP-NDI**	0.589
**Lower back pain**	
**NRS-ODI**	0.160
**NRS-GPE**	0.450
**NRS-RSP**	0.366
**GPE-ODI**	0.381
**GPE-RSP**	0.890
**RSP-ODI**	0.469
**GPE-NRS-RSP**	0.751
**GPE-NRS-ODI**	0.462
**NRS-RSP-ODI**	0.472
**GPE-RSP-ODI**	0.734
**GPE-NRS-RSP-ODI**	0.708

GPE, global perceived effect; NDI, neck disability index; NRS, numeric rating scale; ODI, Oswestry disability index; RSP, residual symptom percentage.

### Agreement between converted numeric rating scale change and residual symptom percentage

**Fig 2:**
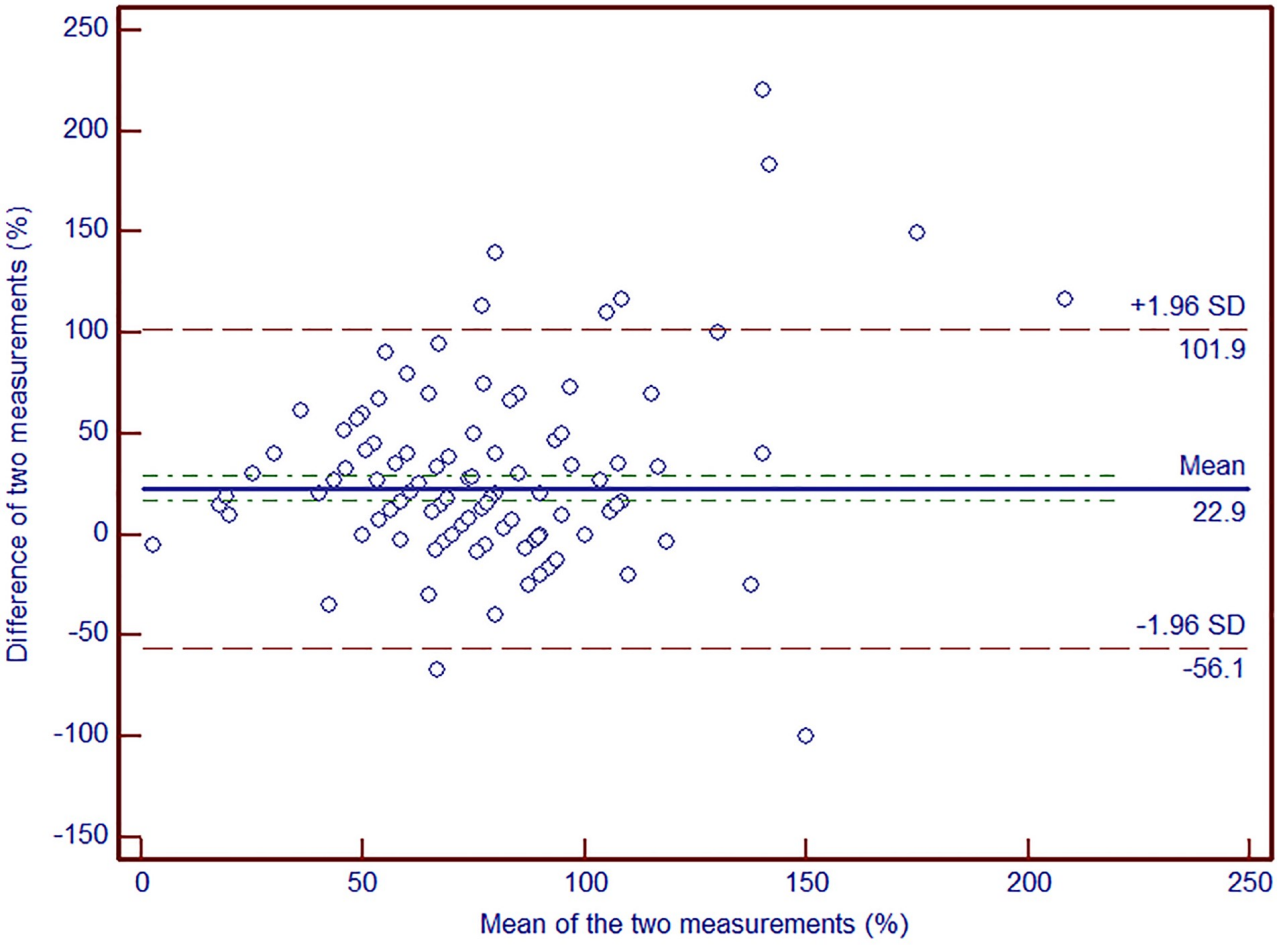
Bland-Altman plot shows agreement between converted numeric rating scale and residual symptom percentage.

The concordance correlation coefficient between the converted numeric rating scale change and residual symptom percentage was 0.26, with a precision of 0.32 and an accuracy of 0.81. The concordance correlation coefficient was 0.60 and 0.51, in neck pain and lower back patient consecutively, when the initial numeric rating scale was higher than 7. It still showed higher concordance (concordance correlation coefficient = 0.53) in all patient with initial numeric rating scale was higher than 7, while concordance correlation coefficient was 0.21 when the initial score was equal to or less than 7 (Table 11). Fig 2 shows a Bland-Altman plot of the two measures.

**Table 11:** Concordance correlation coefficient between converted numeric rating scale change and residual symptom percentage.

	Concordance correlation coefficient	Precision	Accuracy
**Neck pain**			
	0.43	0.51	0.85
**Initial NRS> 7**	0.60	0.72	0.82
**Initial NRS≤ 7**	0.42	0.51	0.82
**Lower back pain**			
	0.16	0.20	0.77
**Initial NRS> 7**	0.51	0.59	0.85
**Initial NRS≤ 7**	0.07	0.12	0.55
**All subject**			
	0.26	0.32	0.81
**Initial NRS > 7**	0.53	0.62	0.85
**Initial NRS≤ 7**	0.21	0.32	0.66

NRS, numeric rating scale.

## Discussion and conclusion

We found that outcome measurements do not always show concordant response for improvement after spine injection. Residual symptom percentage showed good agreement to global perceived effect while numeric rating scale, Oswestry disability index and neck disability index show poor agreement in patient with either neck or low back pain. The concordance correlation coefficient between the residual symptom percentage and the numeric rating scale was very low, although it had higher concordance in patients with severe pain before the spine injection.

Many studies have evaluated the outcomes and efficacy of spine injections for patients with low back pain or neck pain. These studies often use a number of validated measures to assess treatment outcomes. However, most of these validated measurements did not distinguish between methods of treatment or were only performed in patients treated with surgery or rehabilitation programs. Only a few studies have evaluated the responsiveness of outcome measures after spine injections. Surprisingly, most clinicians in the field of spine intervention have been using these outcome measures without skepticism. Tomkins-Lane et al.[9] have evaluated improvements by objectively measuring physical activity after an epidural steroid injection for lumbar spinal stenosis. Their results indicate statistically significant changes in objective measures of performance, but not in pain and functional measurement scores. The mean Oswestry disability index increased after the injection in this study. Shahgholi et al. [12] compared the Patient Reported Outcomes Measurement Information System, a recently developed outcome measure, to other widely used measurement tools used to assess the response after a lumbar transforaminal epidural steroid injection. Comparisons of the new measure to other measurement tools demonstrated that it is responsive and has correlative psychometric properties. Consistent with our experience in clinical practice, the authors describe the challenges and complexities associated with interpreting outcomes when various measurement tools are used.

As demonstrated in our study, each of the outcome measure has a distinct response to treatment. As discussed above, Tomkins-Lane et al. [9] have reported conventional outcome measure does not reflect objectively measured performance. Similar to our study, several studies have compared calculated pain reduction to patient-reported pain reduction to determine the reliability of the outcome measure. Cushman et al. compared the calculated percentage of pain reduction to patient-reported percentage of pain reduction in patients with musculoskeletal pain after a steroid injection. The authors suggest that these two methods not be used interchangeably, as calculated pain reduction tended to overestimate self-reported pain reduction [6]. Theodore et al. compared hourly changes in the numeric rating scale to the global perceived pain reduction after a diagnostic blockade. They found discrepancies between reflecting the pain reduction in two outcome measures, even though there was correlation between these two [8]. We also found discrepancies between the converted numeric rating scale change residual symptom percentage. There were no significant differences in patient demographics, the time interval between injection and follow-up, or previous history of spine injection, similar to what was found in the aforementioned two studies by Cushman et al. and Theodore et al. We also evaluated the initial pain score. However these two measures had higher agreement when the initial pain was severe (numeric rating scale scores higher than 7). Likewise, patients with less pain had smaller converted pain change with poor correlation with the residual symptom percentage. We assume that the numeric rating scale less sensitively represents improvement in patients with mild to moderate pain.

The perception of improvement can be considered an integrated response to patients’ interpretation and judgment of the changes in their own status, which may not include only the relief of pain, but also improvements in emotional state, functional state, and quality of life [13]. However, several studies have questioned the reliability and validity of the global perceived effect and its changes. Kamper et al. [14] have suggested that the global perceived effect may be irrelevant for use as an external criterion of change when evaluating the properties of an outcome measure. This type of patient-reported measure may be influenced by the current health state or the patient’s memory, although this remains to be established [15].

Many previous studies have validated outcome measure used to evaluate chronic back pain, which are useful for evaluating the severity of pain, and disability and functional status before treatment. However in some cases in our study, the Oswestry disability index and numeric rating scale did not show ‘clinically significant changes’, even though the patient reported that they have been improved after spine injection. This finding was confusing when interpreting the results. As we hypothesized, the residual symptom percentage and global perceived effect had high agreement between responses. Although the residual symptom percentage showed a discrepancy in the degree of improvement compared to the converted pain change, there was tendency of pain change between the other two measures. Additionally, in patients with severe pre-treatment pain, there was higher agreement between these measures. Our findings suggest that the residual symptom percentage can be a better representation of improvement after spine injection. We suggest using this measure as a single standard post-treatment outcome measure in clinical practice, especially after spine injection.

Our study has several limitations. First, this was a retrospective study. The time interval between the first visit and the follow-up was different between the patients. This may have affected the responses of the measures based on memory of the initial pain and its progress. Second, this study did not classify patients by chronicity. Acute and chronic pain may have different natural courses of improvement and differences in the pain itself. Third, the severity of spinal degeneration was not considered in our imaging study. In our clinic, candidates for spine injection are divided into two groups. One group includes patients with minor degeneration who are not candidates for surgery. The other group includes surgical candidates who need a spine injection to delay the operation or bridge conservative management for pain control. These differences in patient condition may have also have affected the response to the management. Fourth, there was small number of patient with aggravated pain, which was insufficient to prove statistical significance in symptom aggravation.

In conclusion, among the measures studied, residual symptom percentage showed high agreement to the global perceived effect in response to improvement, while the numeric rating scale and functional outcome measures did not. Comparison of converted numeric rating scale change with residual symptom percentage highlights the fact that the residual symptom percentage better represents improvement than changes in the numeric rating scale in patients, who have lesser pain and recommended for spine injection. Therefore, this result suggest that the residual symptom percentage may be a more practical for clinicians and better represent patients’ improvements after spine injection. Further studies are necessary with detailed pain classification and patient wih wide range of pain change, especially aggravated pain, in study centers with variable clinical background.
